# Correspondence on “Tracking Sources and Dissemination of Indicator Antibiotic Resistance Genes at a Watershed Scale”. Be Aware of DNA Loss during Extraction from Water Samples!

**DOI:** 10.1021/acsestwater.4c00133

**Published:** 2024-09-05

**Authors:** Alexander K.T. Kirschner, Iris Schachner-Groehs, Rita B. Linke, Andreas H. Farnleitner

**Affiliations:** † Medical University Vienna, Institute for Hygiene and Applied Immunology, Water Microbiology, Kinderspitalgasse 15, 1090 Vienna, Austria; ‡ Karl Landsteiner University of Health Sciences, Division Water Quality & Health, Dr. Karl-Dorrek-Strasse 30, 3500 Krems, Austria; § Interuniversity Cooperation Centre Water & Health (www.waterandhealth.at); ∥ Technische Universität Wien, Institute of Chemical, Environmental and Bioscience Engineering, Research Group Microbiology and Molecular Diagnostics, 166/3/5, Gumpendorferstraße 1, 1040 Vienna, Austria

Referring to the recent publication by Tarek et al.,^1^ we want to seize the opportunity to mention the topic of extraction efficiency and loss of DNA (and nucleic acids in general) when analyzing water samples for genetic targets. This is often not sufficiently considered in study conception, sample processing, or evaluation and interpretation of data. In many studies, researchers use standard DNA extraction protocols or DNA extraction kits designed for water samples to determine genetic targets of fecal pollution or antimicrobial resistance (e.g., via qPCR) without considering potential environmental factors that may impact DNA extraction efficiency. Often, as a measure of qPCR performance, the limit of detection (LOD) or quantification (LOQ) of the qPCR is reported.^2^ However, when environmental samples are analyzed, the sample limit of detection (sLOD) or threshold of detection (ToD) should be reported, taking quality control considerations and measures along the whole chain of analysis (WCA)^3^ into account. DNA extraction efficiency is one of the most important sources of bias in this respect, as it can vary significantly,^4^ specifically when inorganic particles are abundant, potentially capturing the free DNA released from the cells during the DNA extraction process.^5^

In the recent publication on antibiotic resistance genes (ARGs) in surface waters of a mixed watershed,^1^ 16S-rRNA-gene copy numbers were reported to range from 2.48 log_10_ (=3 × 10^2^) to 6.36 log_10_ (=2.3 × 10^6^) gene copies per milliliter. When assuming an average of eight 16S-rRNA-gene copies per bacterial cell for a mixed bacterial community,^6^ the lower value would correspond to approximately 40 cells per milliliter of surface river water. The bacterial cell count of highly pristine river water and pristine groundwater usually is on the order of 10^4^–10^5^ cells per milliliter,^7^ so that such a low cell count of 40 cells per milliliter is extremely unrealistic. Unfortunately, the authors did not mention or discuss this topic or the high variability of 16-rRNA-gene copy numbers observed in their paper but stated that “the catchments of the investigated streams are generally characterized by steep slopes, resulting in high water runoff rates and elevated water velocities during storm events”.^1^ On the basis of our own experience,^8^ this leads us to conclude that DNA extraction efficiency might have temporally been impaired, most likely during such storm events resulting in a large amount of inorganic suspended solids in the water. In a highly turbid shallow lake in Austria, we have observed greater impairment of DNA extraction efficiency during storm events, when inorganic sediment particles of the lake bottom were resuspended in the water column.^4,5^ Furthermore, in a very recent study on ARGs in the Danube River, we had samples that showed unrealistically low 16S-rRNA-gene copy numbers (10^2^–10^5^ cells per milliliter). By comparison to total cell counts (TCC) obtained by parallel microscopic examination (10^6^–10^7^ cells per milliliter), we could corroborate that DNA extraction has obviously failed and excluded these samples from the data set.^8^ As the main reason for this failure, we identified a massive stormwater and snowmelt event in a major tributary of the Danube River transporting extremely large loads of inorganic suspended solids. We assume that in both studies DNA released from bacteria during the extraction process (e.g., during bead beating) was captured on the surfaces of the particles (e.g., clay minerals^9^) and thus eluded efficient extraction.^5^ The effect of DNA capture by particles could be aggravated in the presence of metals.^10^ Metal ions such as Al^3–^ and Fe^3–^ bind to clay particles, building a bridge to DNA by complexation with the phosphate group.^10,11^ In fact, in the study on the Danube River, metal concentrations were also massively increased, having potentially contributed to the observed DNA loss during extraction.^8^

If the approach comparing TCC with 16S-rDNA-gene copy numbers would be adopted for the data set presented by Tarek et al.^1^ (Figure S1 of their Supporting Information) and a few thousand cells would be assumed as a realistic TCC for pristine river water (corresponding to ∼10^4^ 16S-rRNA-gene copy numbers), 33 of 132 samples (25%) would exhibit an impaired DNA extraction efficiency. Upon specific examination of the data in their Figure S1, it becomes obvious that for some sites (e.g., sites 2, 7, and 20), clusters of samples occurred that had 16S-rRNA-gene copy numbers markedly lower than those of the other samples, indicating periods of impaired DNA extraction efficiency, likely caused by turbidity events triggered by high water runoff rates and increased water velocities during storm events.^1^ Specifically, such periods could be highly relevant concerning the influx of ARGs from agricultural surfaces or sewer overflows into the rivers, and an impaired DNA extraction could lead to false negative results when this issue is overlooked.

Although stream-gauge and turbidity/total suspended solids data would have been important additional information indicating periods of potential DNA extraction bias in that study,^1^ it is not sufficient to predict DNA extraction problems.^5^ We thus recommend implementing strict quality control concerning DNA extraction in general and specifically when ARG copy numbers are related to 16S-rRNA-gene copy numbers for normalization. The choice of the best-suited quality control method along the WCA depends on the specific research question and molecular analysis method applied.^3^ One straightforward in-line approach is spiking a defined target cell standard as a standard process control strain (DeTaCS, e.g., an *Escherichia coli* strain carrying a target sequence) and calculating the recovery efficiency for each sample (or parallel samples). This was recently proposed in a study on nucleic acid extraction of samples with high turbidity.^5^ Upon analysis of complex community compositions by metagenomic sequencing (e.g., in wastewater surveillance), bacterial mock communities may be applied covering the broad spectrum of bacterial taxa with different cell properties.^12−14^ As a general rule, the spike should come as close as possible to the target. As shown above,^8^ a valuable and simple approach independent of molecular analysis is to determine TCC via epifluorescence microscopy^8^ or flow cytometry,^15^ a parameter that can be used for both quality control of DNA extraction (upon comparison to 16S-rRNA-gene copy numbers) and as a parameter against which ARG copy numbers can be normalized. Recently, normalization of ARG copy numbers to numbers of single-copy genes (equivalent to TCC) instead of 16S-rRNA-gene copy numbers was recommended,^16^ but this approach does not indicate problems with DNA extraction (as both target numbers would be decreased in the case of extraction bias). At least for representative sample subsets and in each extraction batch, specifically when changes in environmental conditions are indicated by relevant parameters, such as turbidity or suspended solids, there is no way around proper quality control during DNA extraction.
